# Long-Term Outcomes of Balloon TACE for HCC: An European Multicentre Single-Arm Retrospective Study

**DOI:** 10.1007/s00270-024-03779-w

**Published:** 2024-07-02

**Authors:** Pierleone Lucatelli, Bianca Rocco, Thierry De Beare, Gontran Verset, Fabio Fucilli, Elio Damato, Alexandro Paccapelo, Lorenzo Braccischi, Makoto Taninokuchi Tomassoni, Ana-Maria Bucalau, Carlo Catalano, Cristina Mosconi

**Affiliations:** 1 Interventional Radiology Unit, Department of Diagnostic Medicine and Radiology, University ‘‘La Sapienza” of Rome, Rome, Italy; 2 Department d’Anesthésie, de Chirurgie, et de Radiologie Interventionnelle, Gustave Roussy, 112 rue Edourad Vaillant, 94805 Villejuif, France; 3 Department of Gastroenterology, Hepatopancreatology and Digestive Oncology C.U.B. Hôpital Erasme, Univerité Libre de Bruxelles, Brussels, Belgium; 4 Radiology Unit, S. De Bellis National Institute of Gastroenterology Research Hospital, Castellana Grotte (BARI), Bari, Italy; 5 Research and Innovation Unit, IRCCS Azienda Ospedaliero-Universitaria di Bologna, Bologna, Italy; 6 Department of Radiology, IRCCS Azienda Ospedaliero-Universitaria di Bologna, Bologna, Italy; 7 Department of Radiology, Alma Mater Studiorum University of Bologna, Bologna, Italy

**Keywords:** Chemoembolisation, Hepatocellular carcinoma, Disease-free survival

## Abstract

**Purpose:**

To report response rates (using mRECIST), overall survival (OS), progression-free survival and local tumour recurrence-free survival (LRFS) of balloon-occluded transarterial chemoembolisation (bTACE) for hepatocellular carcinoma (HCC).

**Materials and Methods:**

Patients from five European centres treated with conventional or drug-eluting microsphere bTACE for HCC were included, and patients already lost to follow-up before 12 months were excluded. Possible factors contributing to LRFS and OS were evaluated with Cox proportional hazards models.

**Results:**

Seventy-three patients were enrolled. The mean number of nodules per patient was 2.07(± 1.68), and the average maximum diameter of the nodules was 37 ± 19.9 mm. The response of the target lesion at 6 months was complete response (CR) in 58.9%, partial response (PR) in 28.8%, stable disease (SD) in 6.8% and progressive disease (PD) in 5.5%. The median follow-up time was 31 months; at the last follow-up, target tumour response was CR in 49.3%, PR in 12.3%, SD in 5.5% and PD 32.9%. Overall response at the last follow-up was CR in 17.8%, PR in 9.6%, SD 2.7% and PD in 69.9% (for new lesions in 37% of patients). Median OS was not reached; mean overall survival was 50.0 months, while median LRFS was 31.0 months. At uni- and multivariable analysis, only tumour maximum diameter was related to LRFS (hazard ratio [HR] = 1.021; 95% CI 1.004–1.038, *P* = 0.015).

**Conclusions:**

bTACE demonstrated high efficacy for HCC, with a complete response in 58.9% of patients, a median local recurrence-free survival of 31.0 months and a mean overall survival of 50.0 months.

**Graphical Abstract:**

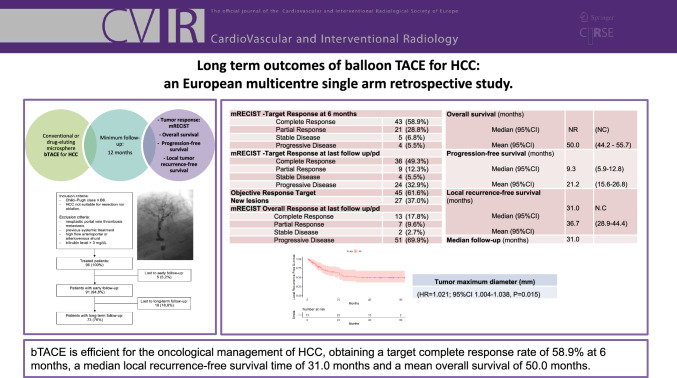

## Introduction

According to the Barcelona Clinic Liver Cancer (BCLC) 2022 update [1], transarterial chemoembolisation (TACE) is indicated for treating HCC in the intermediate stage and in the earlier stage when potentially curative options are not feasible. Despite the non-curative nature of TACE, research efforts in the last decade, as well as technical developments, were aimed at improving treatment response after the initial TACE as complete response (CR) after the first session of treatment has been shown to be related to longer overall survival (OS) [2]. Of the many technical developments, attention was focused on the use of a balloon microcatheter which permits temporary occlusion of the HCC-feeding arteries. Balloon-occluded TACE (bTACE) has been demonstrated to allow pressure gradient-driven embolisation, thus reducing non-target embolisation and allowing a denser deposition of the embolic agent into the tumour [3, 4]. In several studies, this technical advantage showed a trend towards better response rates at the first treatment as compared to standard non-occluded transarterial procedures, either with Lipiodol-based TACE (c-bTACE), drug-eluting microsphere TACE (DEM-bTACE) or radioembolisation [5–9]. In addition, bTACE was seen to have a better tumour response rate, even in HCCs 3–5 cm in diameter which are known to have worse outcomes [10, 11]. Although those results are promising, to date, the majority of the studies on this topic have been single-centre and, without overall survival rates or local recurrence-free survival rates, data which are necessary for evaluating the oncological impact of this technique.

Based on these considerations, the aim of this study was to report the long-term oncological results of bTACE for HCC in a patient population cohort treated in five different European centres, with tumour response evaluated using modified response evaluation criteria in solid tumours (mRECIST) as the primary endpoint, and overall survival, progression-free survival and local tumour recurrence-free survival rates as secondary endpoints.

## Materials and Methods

This study was realised in conformity with the principles of the Declaration of Helsinki and subsequent amendments. Written informed consent for the procedures was obtained from all patients. All personal data were blinded and anonymised in the general database. This study was approved by the Institutional Review Board (protocol number 193/2021/Oss/AOUBo), and the patients were treated with approved diagnostic and therapeutic procedures according to the generally accepted standards of care and were preliminarily discussed in the multidisciplinary tumour board meetings of each centre.

### Patient Selection

**Fig. 1:**
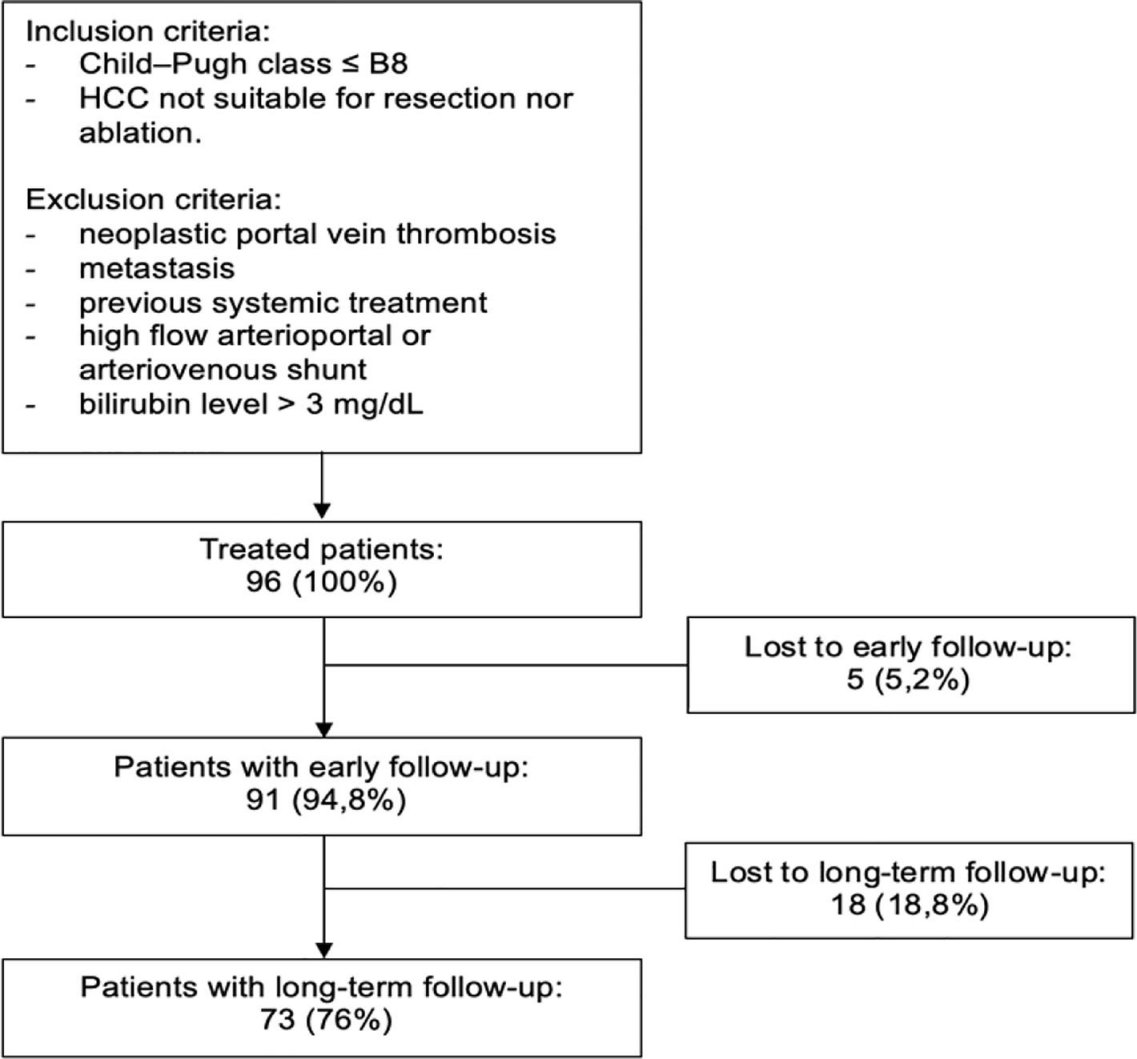
Flowchart summarising the patient selection process

All patients with HCC—treated with bTACE in five European centres (Table 1) (Fig. 1) and with a minimum follow-up of 12 months—were considered in this study. The decision to use a microballoon microcatheter was taken by the operators in each centre while, according to the BCLC classification [1] and the European Association for the Study of the Liver (EASL) clinical practice guidelines [12], either Lipiodol®-based cTACE (balloon-occluded conventional TACE, c-bTACE) or drug-eluting microsphere (balloon-occluded DEM-TACE, DEM-bTACE) was performed. A diagnosis of HCC was obtained using a combination of laboratory tests and imaging examinations, such as contrast-enhanced ultrasound, computed tomography (CT) or magnetic resonance imaging (MRI), according to the current guidelines of the Liver Imaging Reporting & Data System (LI-RADS®) [13].

**Table 1:** Number of patients included in the study divided by centre

Hospital	Patients *n* (%)
IRCCS Azienda Ospedaliero-Universitaria di Bologna,* Italy	9 (12.3%)
Gustave Roussy Cancer Centre,* France	7 (9.6%)
Sapienza University of Rome, Italy	26 (35.6%)
Erasme Hospital Brussels, Belgium	23 (31.5%)
IRCCS De Bellis, Castellana Grotte, Bari, Italy	8 (11.0%)
Overall	73

The inclusion criteria for the treatment were a Child–Pugh class of up to B8 and tumours not suitable for resection or ablation (Table 2). The exclusion criteria were neoplastic portal vein thrombosis, extrahepatic disease, previous systemic treatment, high-flow arterioportal or arteriovenous shunt and a bilirubin level > 3 mg/dL.

**Table 2:** Clinical and radiological characteristics of the patients included in the study

Characteristics	(*n* = 73)
Age (mean ± std. dev.)	67.9 ± 12.1
Gender, *n* (%)	
Male	61 (83.6%)
Female	12 (16.4%)
Etiology, *n* (%)	
Hepatitis C virus (HCV)	27 (37.0%)
Hepatitis B virus (HBV)	12 (16.4%)
Alcohol consumption	19 (26.0%)
Other	15 (20.5%)
Child–Pugh, *n* (%)	
A	56 (76.7%)
B	17 (23.3%)
BCLC stage, *n* (%)	
A	40 (54.8%)
B	32 (43.8%)
C*	1 (1.4%)
Alpha-fetoprotein, ng/mL, median (range)	6.4 (2–54,000)
No. of nodules (mean ± std. dev.)	2.07 ± 1.68
Maximum diameter (mean ± std. dev.)	37.0 ± 19.9
Lipiodol, *n* (%)	18 (24.7%)
Total retreatments, *n* (%)	34 (46.6%)
Transplants, *n* (%)	12 (16.4%)
*Eastern Cooperative Oncology Group (ECOG) scale = 1	

### The Procedure

The bTACE procedure was performed by experienced interventional radiologists (> 10 years of experience), in high-volume European centres. In all cases, a 2.8-Fr microcatheter with an occlusion balloon on the tip (Occlusafe®; Terumo Europe NV, Leuven, Belgium) was used. The balloon, a microballoon, was utilised coaxially in a standard 4- or 5-Fr angiographic catheter. The balloon-occluded arterial stump pressure (BOASP) was measured before and after inflation of the balloon to understand when the microballoon adhered to the vessel wall, thus avoiding over inflation and potential vessel damage. The embolisation was then performed according to the clinical practices of the individual centres, either DEM-bTACE or c-bTACE. The former were performed by injecting drug-eluting microspheres (Lifepearl, Terumo Europe NV, Leuven, Belgium [100 ± 25 µm and 200 ± 50 µm]), pre-loaded with 50 mg of epirubicin per syringe and the latter by injecting a water in oil mixture of epirubicin (75 mg, Farmorubicina®; Pfizer, Latina, Italy) and iodised oil (Lipiodol®; Guerbet, Milan, Italy).

### Oncological Response and Overall Survival

Oncological response was evaluated by imaging (quadriphasic CT or dynamic MRI) 1 month after the procedure and then every 3 months afterwards by the interventional radiologist who performed the procedure in each centre in order to assess the target response at 6 months and the target response at the last follow-up, according to mRECIST [13]. The objective response (OR) rates consisted of the sum of the rates of lesions in CR or PR. Overall disease control (DC) included the rates of patients experiencing complete response, partial response and stable disease.

Overall survival (OS), local recurrence-free survival (LRFS) and progression-free survival (PFS) were calculated.

### Statistical Analysis

Patient demographic and clinical characteristics were reported as frequencies and percentages for the categorical variables, and as mean ± standard deviation or median and range for the continuous variables.

The survival data were computed using the Kaplan–Meier method and were reported as medians or means, together with their 95% confidence intervals (95% CIs). Univariable and multivariable Cox proportional hazard models were used. The hazard ratios were reported together with their 95% CIs. The median follow-up was computed using the reverse Kaplan–Meier method. Patients who underwent liver transplantation were considered censored in the survival analysis.

The p-value was considered significant when less than 0.05 for two-tailed tests. Statistical analysis was carried out using IBM SPSS Statistics software for Windows, version 28.0 (Armonk, NY: IBM Corp).

## Results

### Patient and Treatment Characteristics

Between January 2015 and December 2019, 73 treatment-naïve patients were treated with bTACE in the five centres and were retrospectively analysed in March 2023 (median follow-up of 31.0 months, interquartile range [IQR] 11.3–52.4). The early response rates (up to 6 months) and adverse events of those patients after bTACE have already been published [10]; however, the response and the follow-up after 6 months have been updated for this study.

Mean age was 67.9 ± 12.1 years, with hepatitis C virus (HCV) being the most common cirrhosis aetiology (*n* = 27, 31.0%), followed by hepatitis B virus (HBV) (*n* = 12, 16.4%); 56/73 (76.7%) and 17/73 (23.3%) patients were Child–Pugh A and B, respectively, before the procedure, whereas 40/73 (54.8%) and 32/73 (43.8%) patients were BCLC stages A and B. Only one patient was BCLC stage C having a performance status class 1 according to the Eastern Cooperative Oncology Group. The mean number of nodules per patient was 2.07 ± 1.68 (range 1–9), having an average maximum diameter of 37 ± 19.9 mm (range 13–159); c-bTACE was performed in 18 cases (24.7%) and DEM-bTACE in 55 (75.3%).

### Tumour Response

**Fig. 2:**
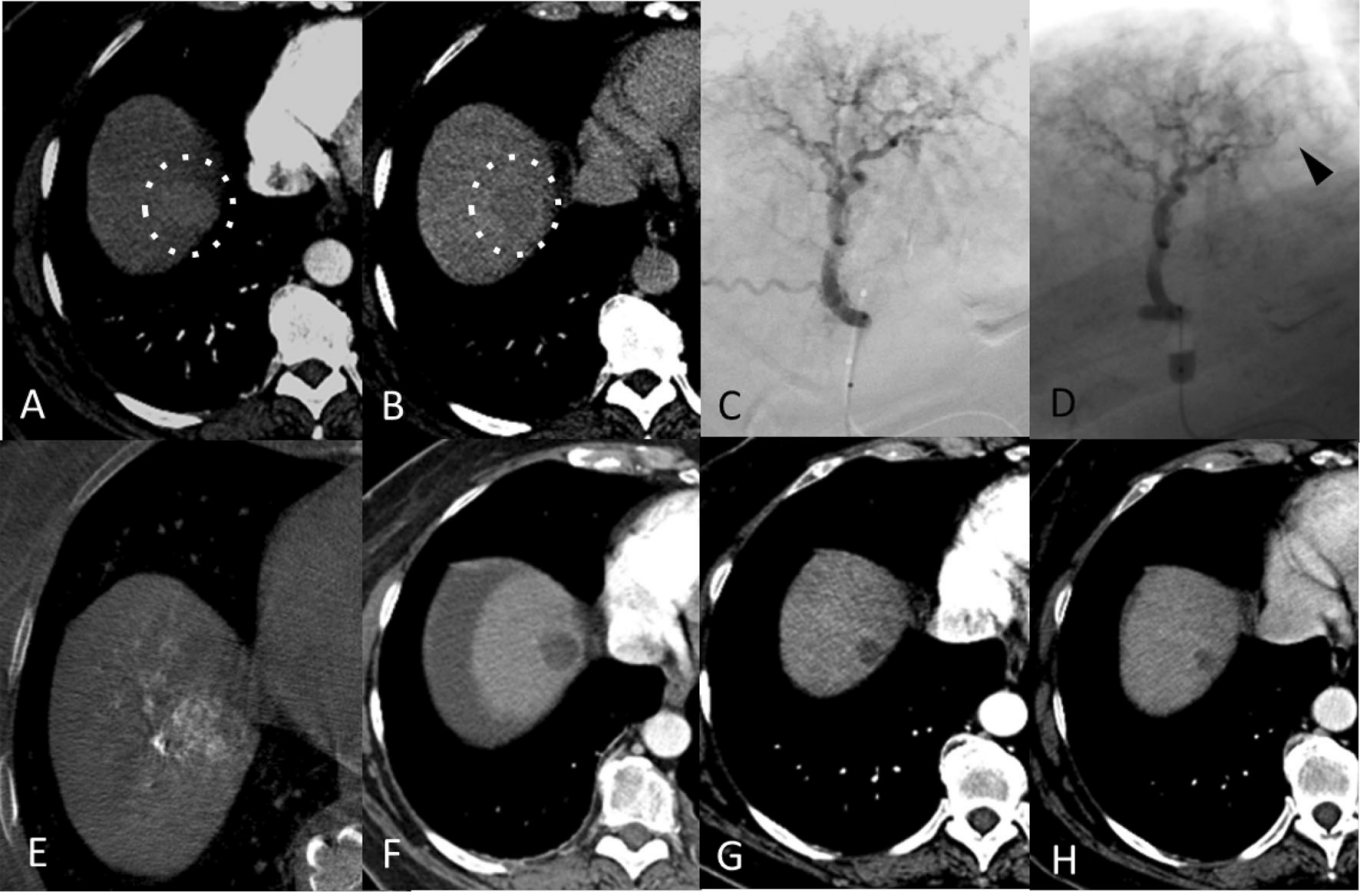
**A** A 64-year-old woman, with alcoholic and HCV cirrhosis complicated by a slightly hypervascular HCC in the arterial phase (**A**, dotted circle) in segment VIII, with late washout (25 mm) (**B**, dotted circle), undergoing a balloon-occluded TACE procedure. The angiography performed with a deflated balloon confirmed the poor hypervascularity of the nodule (**C**) which improved after balloon inflation (**D**, arrow head). The unenhanced cone beam CT performed at the end of the bTACE showed the good deposition of the particles within the nodule (**E**). One-month post-procedure CT arterial phase follow-up showed a complete response (**F**) sustained at 12 months (**G**) and at the last follow-up available, carried out at 24 months (**H**)

Evaluation of the response of the targeted tumours at 6 months was CR in 43 patients (58.9%), PR in 21 patients (28.8%), SD in 5 (6.8%) and PD in 4 (5.5%). At the last follow-up, 36/73 patients (49.3%) had a CR, 9 patients a PR (12.3%), 4 patients SD (5.5%) and 24 patients (32.9%) had PD of the target lesion, yielding a 61.6% objective response rate and a 67.1% disease control rate (Fig. 2).

The overall response rate at the last follow-up was complete response in 13 patients (17.8%), partial response in 7 patients (9.6%), stable disease in 2 patients (2.7) and progressive disease in 51 patients (69.9%) (Table 3). New lesions were observed in 27 patients (37.0%). Seven (9.6%) patients underwent completion of locoregional treatment within the first 6 months on target lesions (Table 4); treatments after 6 months on target and non-target lesions are described in Table 4.

**Table 3:** Clinical and radiological outcomes of the patients treated using survival rate and Modified Response Evaluation Criteria in Solid Tumours (mRECIST) criteria

Outcomes	(*n* = 73)
Overall survival, months	
Median (95% CI)	*Not reached* (*not calculable*)
Mean (95% CI)	50.0 (44.2—55.7)
Progression-free survival, months	
Median (95% CI)	9.3 (5.9–12.8)
Mean (95% CI)	21.2 (15.6–26.8)
Local recurrence-free survival, months	
Median (95% CI)	31.0 (*not calculable*)
Mean (95% CI)	36.7 (28.9–44.4)
Median follow-up (months)	31.0
Patients with local recurrence, *n* (%)	24 (32.9%)
mRECIST target response at 6 months	
Complete response	43 (58.9%)
Partial response	21 (28.8%)
Stable disease	5 (6.8%)
Progressive disease	4 (5.5%)
mRECIST target response at last follow-up/pd	
Complete response	36 (49.3%)
Partial response	9 (12.3%)
Stable disease	4 (5.5%)
Progressive disease	24 (32.9%)
Disease control target	49 (67.1%)
Objective response target	45 (61.6%)
New lesions	27 (37.0%)
mRECIST overall response at last follow-up/pd	
Complete response	13 (17.8%)
Partial response	7 (9.6%)
Stable disease	2 (2.7%)
Progressive disease	51 (69.9%)

**Table 4:** Retreatments occurred during the entire follow-up on target and non-target lesions

Typology of retreatments	Target lesions	Non-target lesions	Total
Before 6 months			
TACE	7/73 (9.6%)	7/73 (9.6%)	14/73 (19.2%)
After 6 months, during the entire follow-up period			
Systemic therapy	0/73 (0%)	3/73 (4.1%)	3/73 (4.1%)
Radiofrequency	1/73 (1.4%)	0/73 (0%)	1/73 (1.4%)
Radioembolisation	2/73 (2.8%)	0/73 (0%)	2/73 (2.8%)
TACE	6/73 (8.2%)	6/73 (8.2%)	12/73 (16.4%)
TACE + Radioembolisation	1/73 (1.4%)	0/73 (0%)	1/73 (1.4%)
TACE + Radiofrequency	1/73 (1.4%)	0/73 (0%)	1/73 (1.4%)
Total retreatments	18/73 (24.7%)	16/73 (21.9%)	34/73 (46.6%)

### Overall Survival Rate

**Fig. 3:**
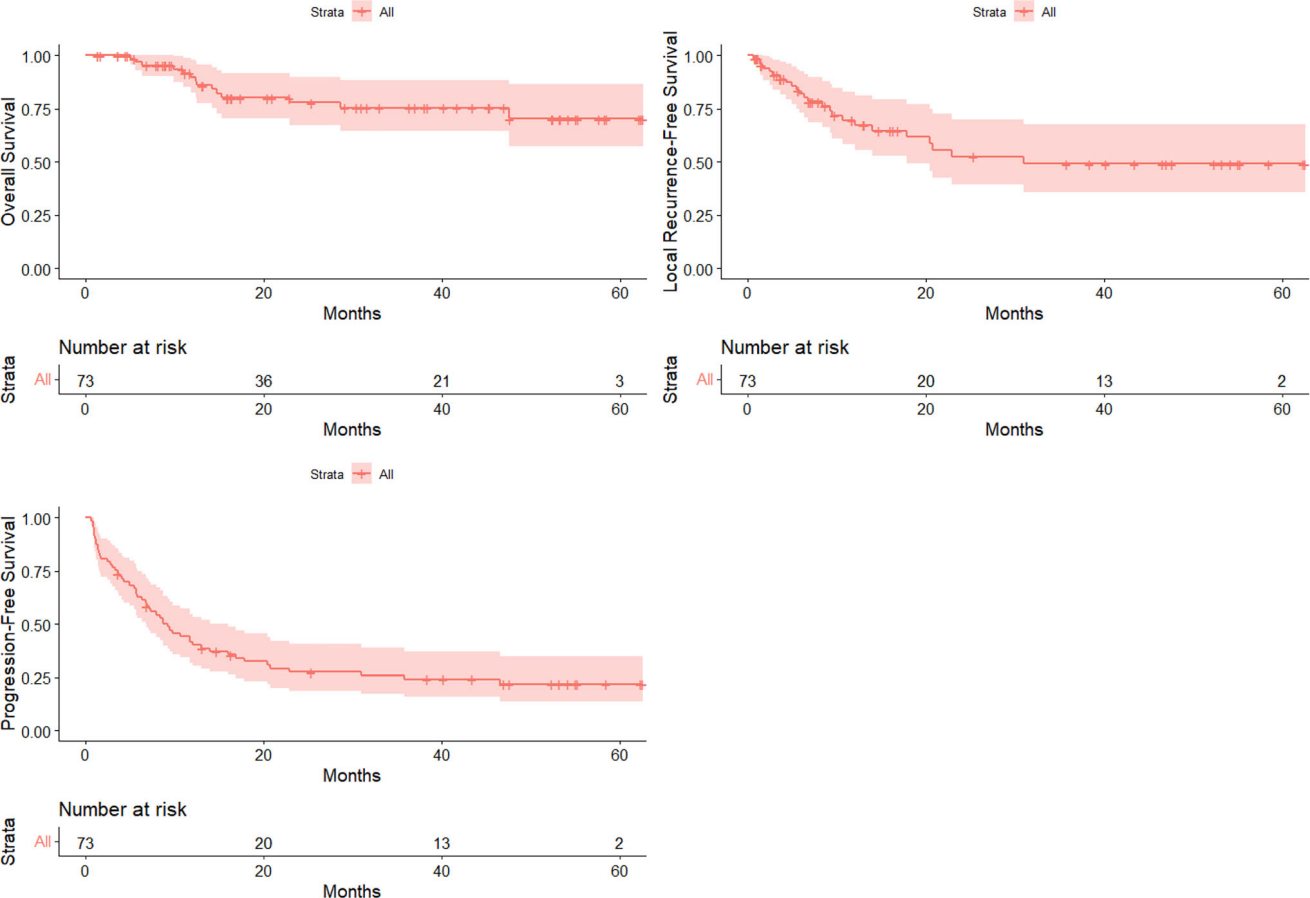
Overall, local recurrence-free and progression-free survival curves

The mean projected OS was 50.0 months (95% CI 44.2–55.7), namely 51.4 (95% CI 44.5–58.3) months for patients BCLC A and 42.1 (95% CI 33.3–58.3) for patients BCLC B, while median overall survival was not reached (Fig. 3).

The median LRFS time was 31.0 months (Fig. 3). Of the 24 patients with local recurrence, a mean recurrence time of 9.4 months and a median of 6.6 months were observed. The median PFS was 9.3 months (Table 3 and Fig. 3).

After uni- and multivariable analysis, only the dimension of the diameter seemed to be related to local recurrence-free survival (HR = 1.021; 95% CI 1.004–1.038, *P* = 0.015). The hazard ratio reported referred to the correlation between the linear increases of 1 mm in maximum diameter with an average 2% increase in risk of local recurrence per additional millimetre.

No variable appeared to be related to progression-free survival or overall survival (Table 5).

**Table 5:** Local recurrence-free survival

Local recurrence-free survival	Univariable					Multivariable				
	HR	95% CI			*P* value	HR	95% CI			*P* value
Age (years)	1.004	0.969	–	1.041	0.821					
Gender = Female	0.962	0.285	–	3.249	0.950					
Child–Pugh = B	1.471	0.608	–	3.561	0.392					
Lipiodol	1.735	0.769	–	3.913	0.184					
No. of nodules	1.104	0.897	–	1.359	0.349					
Maximum diameter (mm)	1.021	1.004	–	1.038	0.015	1.021	1.004	–	1.038	0.015

HR, hazard ratio and 95% CI, 95% confidence intervals

## Discussion

This longitudinal long-term follow-up of bTACE in HCC (median diameter 37 mm) demonstrated a complete response rate at 6 months of 58.9%, a median LRFS of 31 months and an OS of 50 months.

The mean OS of 42 months observed in the BCLC B patients in the present study was longer than the 2.5 years expected for the BCLC B population, while the 50 months of OS in the present study was almost in line with the 5-year OS expected for BCLC A patients.

Complete response rates of 58.9% at 6 months and of 49.3% at the last available follow-up (median 31 months), a major findings in this study as achieving a CR after initial treatment [2], as well as maintaining it [14], have been demonstrated to be a strong predictor of longer OS. The CR rate reported was higher than that in the previously published literature regarding cTACE and DEM-TACE, demonstrating a clinical advantage in using a microballoon catheter. Notably, a recent large study by Peng et al. [15], which enrolled 669 patients treated with both cTACE and DEM-TACE, achieved a CR rate of 22.3% at the first follow-up versus the 58.9% observed at 6 months in the present series. The outcomes in the present study were also similar in terms of response rates to those reported in the most recent and largest study report on standard DEM-TACE [16], even though with a longer timeframe (6 months vs. 1 month) and with larger nodules. In fact, Veloso Gomez et al. reported a retrospective multicentric pooled analysis of different series from different European centres using DEM-TACE in 580 HCC patients. Presented data did not displayed median target nodule diameter but reported the “sum of the lesions diameter” (53.1 ± 33.3 mm) with a mean number of lesions of 2.1 ± 1.5, multifocal in 61% of cases, thus leading to lesions smaller to the ones treated in this series. Veloso Gomez et al. reported a best CR rate of 60.14%, which is among the highest reported for TACE, similar to the CR rates obtained at the 6-month timeframe for larger lesions in the present experience with bTACE.

The response rates of the targeted tumours were in line with other previously published smaller size series on bTACE [17, 18]. Shirono et al. reported an LRFS of 39.3 months in a population of 25 patients with 45 HCCs having a mean diameter of 21-mm smaller than those treated in the present series. Moreover, Chu et al. [18], by performing a propensity score matching for 32 pairs of patients treated with bTACE and cTACE, demonstrated a higher initial complete response rate of bTACE over non-occluded TACE, as well as a longer time to local tumour progression (27 vs. 13 months) in lesions greater than 3 cm. These data represent a glimpse of the worldwide experience (Korea, Japan and Europe) regarding this technique, supporting the evidence that bTACE is capable of increasing the rate of initial complete response as well as determining a longer LRF response for non-occluded procedures. This could be the result of a pressure gradient-driven embolisation which potentially leads to a denser accumulation of the embolic agent with consequently better coverage of the area being treated as well as encompassing potential HCC satellite.

The multivariable analysis demonstrated how the unique predictor factor for a lower local recurrence-free survival rate was target lesion diameter. This result was in line with what had previously been reported by Chu et al. [18] who had identified a higher local tumour progression rate in lesions > 3 cm.

Analysing the retreatment rate for target lesion emerges that after initial bTACE during the entire follow-up, the majority of the patients (75.4%) did not need further treatment sessions, whereas only in 24.6% (18/73), adjunctive locoregional treatment was needed to control relapse/progression of the target lesion. This is relevant if considering the initial mean tumoral diameter of 37 mm [18]. Also, further therapies on target and not-target lesions demonstrate that bTACE does not affect patient liver function and allows further treatment in case of progression of disease.

The long-term follow-up of this cohort demonstrated that the results obtained in the first session of treatment remained stable over a long follow-up; this was achieved despite the bTACE procedures being performed in various countries which used different embolisation platforms, suggesting how these results were reproducible and related to the use of a microballoon. On the basis of this, it seems reasonable to affirm that bTACE could outperforms non-occluded TACE oncological performance. Additional studies are needed to demonstrate this trend of better oncological performance in a randomised fashion.

The limitations of the study are its retrospective nature with almost 25% of the initially treated patients excluded from this long-term analysis as they were already lost to follow-up before 12 months, the absence of a control group—which had been included in the Authors’ previous study, but was not included in this study due to the absence of an equivalent adequate long-term follow-up, and the relatively small population included.

## Conclusion

This multicentric European long-term analysis of the oncological results of bTACE for HCC indicated its efficacy, obtaining a target complete response rate of 58.9% at 6 months, a median local recurrence-free survival time of 31.0 months and a mean overall survival of 50.0 months.
